# Determination of an Interaction Network between an Extracellular Bacterial Pathogen and the Human Host

**DOI:** 10.1128/mBio.01193-19

**Published:** 2019-06-18

**Authors:** Brad Griesenauer, Tuan M. Tran, Kate R. Fortney, Diane M. Janowicz, Paula Johnson, Hongyu Gao, Stephen Barnes, Landon S. Wilson, Yunlong Liu, Stanley M. Spinola

**Affiliations:** a Department of Microbiology and Immunology, Indiana University School of Medicine, Indianapolis, Indiana, USA; b Department of Medicine, Indiana University School of Medicine, Indianapolis, Indiana, USA; c Department of Pediatrics, Indiana University School of Medicine, Indianapolis, Indiana, USA; d Department of Medical and Molecular Genetics, Indiana University School of Medicine, Indianapolis, Indiana, USA; e Department of Pharmacology and Toxicology, University of Alabama at Birmingham, Birmingham, Alabama, USA; f Targeted Metabolomics and Proteomics Laboratory, University of Alabama at Birmingham, Birmingham, Alabama, USA; g Department of Biostatistics, Indiana University School of Medicine, Indianapolis, Indiana, USA; h Department of Pathology and Laboratory Medicine, Indiana University School of Medicine, Indianapolis, Indiana, USA; University of Würzburg; University of Connecticut; Emory University School of Medicine

**Keywords:** dual RNA-seq, *Haemophilus ducreyi*, human infection model, interactome, metabolome

## Abstract

A major gap in understanding infectious diseases is the lack of information about molecular interaction networks between pathogens and the human host. Haemophilus ducreyi causes the genital ulcer disease chancroid in adults and is a leading cause of cutaneous ulcers in children in the tropics. We developed a model in which human volunteers are infected on the upper arm with H. ducreyi until they develop pustules. To define the H. ducreyi and human interactome, we determined bacterial and host transcriptomic and host metabolomic changes in pustules. We found that *in vivo* H. ducreyi transcripts were distinct from those in the inocula, as were host transcripts in pustule and wounded control sites. Many of the upregulated H. ducreyi genes were found to be involved in ascorbic acid and anaerobic metabolism and inorganic ion/nutrient transport. The top 20 significantly expressed human pathways showed that all were involved in immune responses. We generated a bipartite network for interactions between host and bacterial gene transcription; multiple positively correlated networks contained H. ducreyi genes involved in anaerobic metabolism and host genes involved with the immune response. Metabolomic studies showed that pustule and wounded samples had different metabolite compositions; the top ion pathway involved ascorbate and aldarate metabolism, which correlated with the H. ducreyi transcriptional response and upregulation of host genes involved in ascorbic acid recycling. These data show that an interactome exists between H. ducreyi and the human host and suggest that H. ducreyi exploits the metabolic niche created by the host immune response.

## Abstract (precis)

Dual RNA sequencing (RNA-seq) offers the promise of determining an interactome at a transcriptional level between a bacterium and the host but has yet to be done on any bacterial infection in human tissue. We performed dual RNA-seq and metabolomics analyses on wounded and infected sites following experimental infection of the arm with H. ducreyi. Our results suggest that H. ducreyi survives in an abscess by utilizing l-ascorbate as an alternative carbon source, possibly taking advantage of host ascorbic acid recycling, and that H. ducreyi also adapts by upregulating genes involved in anaerobic metabolism and inorganic ion and nutrient transport. To our knowledge, this is the first description of an interaction network between a bacterium and the human host at a site of infection.

## INTRODUCTION

A major gap in understanding infectious diseases is the lack of information about molecular interaction networks, also known as interactomes, between pathogens and the human host. Dual RNA sequencing (RNA-seq) allows unbiased coexpression analyses of human and pathogen transcriptomes without first separating their respective RNAs and offers the potential of determining an interactome at a transcriptional level at sites of human infection (1). While dual RNA-seq has been utilized for bacterial pathogens in human cell culture models (2–5) and animal infection models (6–8), determination of an interactome during a bacterial infection of humans has yet to be accomplished. As a complement to transcriptomics, metabolomics allows analysis of the physiological state of infected sites via direct functional readouts (9). Combinations of these systems biology approaches will allow a better understanding of the interplay between a pathogen and its host.

Haemophilus ducreyi is a Gram-negative, facultative anaerobe and the causative agent of chancroid—a sexually transmitted genital ulcer disease that facilitates the transmission of human immunodeficiency virus type 1 (10). In addition to causing chancroid, H. ducreyi is a leading cause of non-sexually transmitted cutaneous ulcers (CU) in children in yaws-endemic areas in the tropics (11–14). Although mass administration of azithromycin initially decreased the prevalence of H. ducreyi-associated CU in areas of endemicity (14), the organism was not eradicated (15), possibly due to environmental reservoirs containing H. ducreyi (16, 17). The failure of antibiotics to eradicate CU caused by H. ducreyi highlights a need to understand the interplay between H. ducreyi and the human host.

To study the biology of H. ducreyi, we developed a model in which healthy adult volunteers are infected on the upper arm via puncture wounds with genital ulcer strain 35000HP (HP; human passaged) until they develop pustules (18). Whole-genome sequencing shows that ∼70% of CU strains and 35000HP diverged from a common ancestor ∼180,000 years ago and differ from each other by only ∼400 single nucleotide polymorphisms, most of which are synonymous (19, 20). Thus, this model is highly relevant to CU. During experimental infection, fibrin and collagen deposit in the wounds followed by trafficking of macrophages and polymorphonuclear cells (PMNs) to form micropustules in the dermis and epidermis (21, 22). Within 2 days of infection, the micropustules become an abscess due to accumulation of PMNs (21, 22). Below the abscess is a macrophage collar, while effector memory and central memory CD4 and CD8 T cells, NK cells, Langerhans cells, and myeloid dendritic cells infiltrate the dermis (22–26). In both experimental and natural infections, H. ducreyi associates with both macrophages and PMNs but is extracellular, as these immune cells fail to ingest the pathogen (22, 27). Thus, H. ducreyi must evade phagocytosis and adapt to the nutrient-poor, anaerobic environment of the abscess, which includes serum, activated complement, oxidative products, and antimicrobial peptides, in order to survive.

Using RNA-seq, we previously showed that H. ducreyi gene expression in experimental pustules is distinct from historical data sets obtained from different phases of *in vitro* growth (28). Compared to mid-log-phase cells, which are used to infect volunteers, H. ducreyi upregulates only a few virulence determinants required for progression to the pustular stage of disease. Instead, the organism upregulates pathways *in vivo* that are involved with uptake of alternative carbon sources, nutrient transport, and anaerobic metabolism (28), suggesting that H. ducreyi primarily alters its gene expression to adapt to the unique metabolic niche shaped by the host immune response in the abscess. As this pilot study utilized convenience samples obtained from volunteers who participated in mutant versus parent trials and who were not sham inoculated, we could not determine which host genes were differentially regulated at infected sites. However, the pilot study showed that determination of an interactome between the human host and H. ducreyi was feasible.

In the present study, we experimentally infected human volunteers with H. ducreyi and profiled the transcriptomes of infected and wounded sites using dual RNA-seq. We also determined changes in the environment of infected and wounded sites using nontargeted metabolomics. We sought to determine correlations between bacterial and host gene expression and between differential gene expression and the metabolome at infected sites. To our knowledge, this is the first determination of a bacterium-host interaction network and its relationship to the metabolome during a human infection.

## RESULTS

### Experimental H. ducreyi infection of human volunteers.

To determine if an interaction network exists between H. ducreyi and the human host and whether host transcriptional changes correlate with the metabolome, we inoculated 8 volunteers (3 men, 5 women; 5 whites, 2 blacks, 1 native American; 40.3 ± 11.4 years old) with 144 ± 7 CFU of 35000HP at 3 sites and at 1 site with a buffer control in 3 iterations. Five of the volunteers formed at least 1 pustule and underwent 6-mm-diameter excisional punch biopsy sampling of infected and wounded sites 6 to 8 days later. Three men (identified here as patients 462, 465, and 466) contributed 2 pustules for both RNA-seq and metabolomics; 1 woman (467) contributed 1 pustule for RNA-seq; another woman (468) contributed 1 pustule for metabolomics. If a volunteer contributed pustules for both metabolomics and transcriptomics, the biopsy sample from their wounded site was divided in half at the bedside before processing.

### Global gene expression analyses of H. ducreyi and the human host.

**FIG 1:**
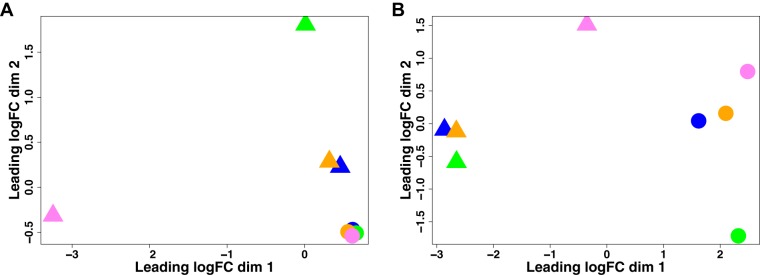
Multidimensional scaling from infected (▲) and control (●) samples. Colors indicate samples corresponding to each volunteer as follows: 462 = blue, 465 = green, 466 = orange, and 467 = violet. (A) Separation of bacterial transcripts in infected sites and the inocula (*P = *0.030 [PERMANOVA]). (B) Separation of host transcripts in infected and wounded sites (*P = *0.033 [PERMANOVA]). logFC, log fold change.

We isolated RNA from infected tissue, wounded tissue, and the H. ducreyi inocula used to infect the subjects and performed dual RNA-seq to identify the transcriptomes of both H. ducreyi and the host. Read sizes of infected samples measured from 418 to 475 million with 0.003% to 0.15% of genes mapped to the H. ducreyi genome and 98.1% to 98.7% of genes mapped to human genome; coverage for H. ducreyi ranged from 1.14-fold to 55-fold (Table 1). From wounded sites, read sizes measured from 85 to 106 million (Table 1) and from the inocula averaged ∼63 million (data not shown). Multidimensional scaling (MDS) of the H. ducreyi transcriptional profile *in vivo* versus that of H. ducreyi from the inocula showed separation of each profile (*P = *0.030 by permutational multivariate analysis of variance [PERMANOVA]; Fig. 1A), confirming our previous data (28). MDS also showed separation of host transcripts in infected and wounded sites in dimension 1 and by host in dimension 2 (*P = *0.033; Fig. 1B). Values representing Pearson correlation coefficients (*r*) corresponding to differences in levels of H. ducreyi gene expression ranged from 0.95 to 0.97 between the inocula and from 0.91 to 0.95 between the infected sites, while *r* values representing human gene expression ranged from 0.96 to 0.98 between the wounded sites and from 0.93 to 0.98 between the infected sites (see Fig. S1 in the supplemental material).

**TABLE 1:** RNA-seq read statistics from biopsy samples and wounds

Subject no.^*a*^	Library size (no. of reads) × 10^6^	No. of H. ducreyi reads^*b*^ × 10^6^	% H. ducreyi reads	H. ducreyi fold coverage	No. of human reads^*b*^ × 10^6^	% human reads
462	418.1	0.537	0.15	47	412.0	98.5
462c	90.3	NA	NA	NA	88.8	98.2
465	431.8	0.046	0.012	4	423.8	98.1
465c	106.1	NA	NA	NA	103.7	97.7
466	475.0	0.622	0.15	55	468.7	98.7
466c	84.7	NA	NA	NA	83.1	98
467	436.8	0.013	0.003	1.14	429.2	98.3
467c	92.6	NA	NA	NA	90.6	97.9

a The indicated numbers were used to identify volunteers with infections corresponding to each infected and wounded (c) site.

b Data represent the number of reads that mapped to 35000HP genes or hg38 genes. NA, not applicable.

10.1128/mBio.01193-19.1 FIG S1 Heat maps of (A) H. ducreyi and (B) human genes showing Pearson correlation coefficients (r) of results of comparisons between volunteers at infected sites (patients 462, 465, 466, and 467) and (A) inocula or (B) wounded sites (462c, 465c, 466c, and 467c), respectively. Pearson correlation coefficients were calculated after removal of genes expressed at low levels and transformation of counts to counts per million. Download FIG S1, PDF file, 0.5 MB. Copyright © 2019 Griesenauer et al. 2019 Griesenauer et al. This content is distributed under the terms of the Creative Commons Attribution 4.0 International license.

### H. ducreyi differential gene expression profile.

**FIG 2:**
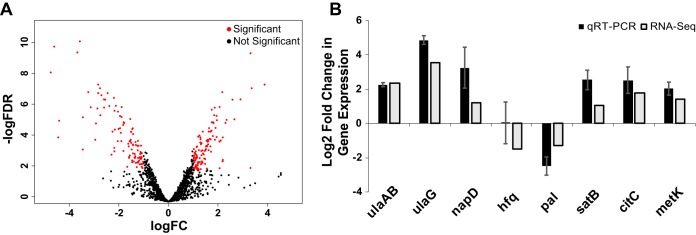
(A) Volcano plot of levels of H. ducreyi transcript expression from four infected versus inoculum samples. Red data indicate genes showing differential expression based on cutoffs of an absolute log_2_ fold change value of >1 and a false-discovery rate of <0.01. A total of 218 genes were differentially expressed. (B) qRT-PCR validation of data from eight selected H. ducreyi genes defined as differentially expressed by RNA-Seq. Target gene expression levels were normalized to that of *dnaE*. Data represent mean ratios of results from three biopsy specimens divided by values corresponding to expression from an inoculum sample used for the RNA-Seq study.

We identified differentially expressed H. ducreyi genes by using cutoff values of absolute log_2_ fold change of >1 and false-discovery rate (FDR [*q*]) of <0.01. A positive fold change indicates higher expression in the infected samples, and a negative fold change indicates higher expression in the control samples. Compared to the inocula, *in vivo* H. ducreyi differentially expressed genes (DEGs) totaled 218 (Fig. 2A), consisting of 81 monocistronic and 80 polycistronic operons. Of those 218 DEGs, 113 were upregulated and 105 were downregulated *in vivo* compared to the inocula (see Table S1 in the supplemental material). We chose eight DEGs for validation using reverse transcription-quantitative PCR (qRT-PCR) (primer list found in Table S2). As the levels of expression of *dnaE* did not differ between infected and inoculum samples (log_2_ fold change = 0.2, *q* = 0.74), we used *dnaE* as a reference and confirmed differential expression of 7/8 DEGs identified by RNA-seq (Fig. 2B). Fold changes in expression of the tested genes determined by qRT-PCR correlated strongly with the fold changes in expression of the same genes as determined by RNA-seq with a coefficient of determination of 0.79.

10.1128/mBio.01193-19.3 TABLE S1 Differentially expressed H. ducreyi genes in infected sites versus the inocula. Download Table S1, XLSX file, 0.03 MB. Copyright © 2019 Griesenauer et al. 2019 Griesenauer et al. This content is distributed under the terms of the Creative Commons Attribution 4.0 International license.

10.1128/mBio.01193-19.4 TABLE S2 H. ducreyi primers used in this study. Download Table S2, DOCX file, 0.01 MB. Copyright © 2019 Griesenauer et al. 2019 Griesenauer et al. This content is distributed under the terms of the Creative Commons Attribution 4.0 International license.

Using the Kyoto Encyclopedia of Genes and Genomes (KEGG), we classified the H. ducreyi DEGs into functional categories. Pathways dominated by upregulated DEGs included carbohydrate metabolism, secretion, signal transduction, transporters, replication and repair, and translation; pathways dominated by downregulated DEGs corresponded to nucleotide metabolism, ribosome biogenesis, chaperones, and many poorly characterized proteins (Table 2). Specifically, genes or operons (referred to here as genes for simplicity) involved in l-ascorbate and aldarate metabolism (*ulaABCD* and *ulaGREF*) and in manganese (*yfeAB*), iron (*yfeCD*), and glycerol (*glpF*) transport were upregulated. Genes involved in citrate metabolism (*citCDEFG*), periplasmic nitrate reductase genes (*napFDAGHBC*), and genes involved in anaerobic respiration and fermentation (*dcuB2* and *focA*) were upregulated (Table S1). As all of these genes are generally upregulated under anaerobic conditions (29–33), these data suggest a shift in H. ducreyi metabolism to anaerobic growth at infected sites.

**TABLE 2:** Summary of KEGG classification of H. ducreyi DEGs

KEGG term	Upregulated	Downregulated
Metabolism		
Amino acid	2	3
Carbohydrate	10	3
Cofactor and vitamin	1	1
Energy	7	4
Glycan	1	3
Nucleotide	1	8
Protein	2	2
Terpenoids and polyketides	0	1
Total	24	25

Cell processing and signaling		
Cell division	0	1
Chaperones	0	4
Folding, sorting, and degradation	1	3
Membrane transport	5	4
Secretion	15	2
Signal transduction	6	2
Transporters	16	9
Total	43	25

Genetic information processing		
Replication and repair	6	1
Ribosome biogenesis	1	4
Transcription	3	1
Translation	12	5
Total	22	11

Poorly characterized		
Hypothetical	16	26
Uncharacterized conserved	8	18
Total	24	44

Total no. of differentially expressed genes	113	105

Next, we grouped H. ducreyi genes into sets based on KEGG identifiers containing 141 different manually curated gene sets (Table S3). Due to the small size of the H. ducreyi genome (∼1.7 Mbp and ∼2,000 genes), many gene sets contained <10 genes (75/141 or 53.1%). To reduce the potential for errors associated with performing statistical tests on gene sets having <10 genes, we focused only on gene sets that were considered significantly different in multiple tests. Using gene set enrichment analysis (GSEA) and coincident extreme ranks in numerical observations (CERNO) tests, ascorbate and aldarate metabolism and bacterial motility proteins were the only two groups found to be significantly upregulated between the biopsy samples and inocula in both tests. Eight of the 10 genes in the bacterial motility proteins set were from the *flp-tad* operon, which is important for microcolony formation and is required for pustule formation in humans (Table S1) (34, 35). No downregulated gene sets reached statistical significance.

10.1128/mBio.01193-19.5 TABLE S3 H. ducreyi gene set list defined by KEGG classification. Download Table S3, XLSX file, 0.1 MB. Copyright © 2019 Griesenauer et al. 2019 Griesenauer et al. This content is distributed under the terms of the Creative Commons Attribution 4.0 International license.

### Human differential gene expression profile.

**FIG 3:**
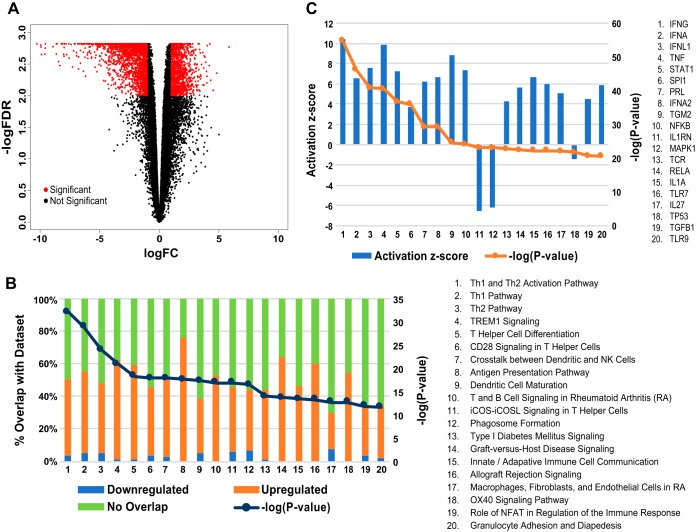
(A) Volcano plot of levels of human transcript expression from four infected versus wound samples. Red data indicate genes showing differential expression based on cutoffs of absolute log_2_ fold change values of >1 and false-discovery-rate values of <0.01. A total of 2,880 genes were differentially expressed. (B) Canonical pathway analysis of DEGs using IPA. The top 20 significantly altered pathways in infected versus wound samples are shown. (C) Upstream regulator analysis of DEGs using IPA. The top 20 upstream transcriptional regulators in infected versus wound samples are shown.

Using an absolute log_2_ fold change cutoff of >1 and a false-discovery-rate cutoff of <0.01, human DEGs totaled 2,880 (Fig. 3A): 1,873 were upregulated and 1,007 were downregulated in the infected sites versus the wounded sites (Table S4). We used Ingenuity pathway analysis (IPA) and GSEA to group DEGs to better understand which host pathways were being affected during experimental H. ducreyi infection. Of the top 20 significantly different pathways identified using IPA and GSEA, the latter using Gene Ontology (GO) terms as our gene set database, all involved the upregulation of genes relating to the immune response (Fig. 3B; see also Fig. S2 and Table S5 in the supplemental material). This was confirmed statistically using the CERNO test (data not shown). While many of the top pathways involved T cell activation (i.e., Th1 and Th2 activation pathway, Th1 pathway, Th2 pathway, and T helper cell differentiation pathway), some pathways also focused on the innate response (i.e., TREM1 [triggering receptor expressed on myeloid cells 1] signaling, cross talk between dendritic cells and NK cells, and phagosome formation). We also identified upstream regulators of the host DEGs using IPA (Fig. 3C; see also Table S6). Upstream regulator analysis predicts transcriptional regulators, defined as representing any molecule that can affect the expression of other genes. A positive Z-score indicates activation, and a negative Z-score indicates inhibition. Most of the activated regulators are involved in promoting the immune response. The two genes encoding regulators with significant (Z-score greater than 2 or less than −2) negative Z-scores, *Il1rn* and *Mapk1*, have been implicated in suppressing the immune response via inhibiting interleukin-1 (IL-1) and gamma interferon (IFN-γ) signaling, respectively.

10.1128/mBio.01193-19.2 FIG S2 (A) Upregulated and (B) downregulated pathway analysis of human DEGs using GSEA. DEGs were classified into gene sets by gene ontology terms. Download FIG S2, PDF file, 0.01 MB. Copyright © 2019 Griesenauer et al. 2019 Griesenauer et al. This content is distributed under the terms of the Creative Commons Attribution 4.0 International license.

10.1128/mBio.01193-19.6 TABLE S4 Differentially expressed human genes in infected versus wounded sites. Download Table S4, XLSX file, 0.2 MB. Copyright © 2019 Griesenauer et al. 2019 Griesenauer et al. This content is distributed under the terms of the Creative Commons Attribution 4.0 International license.

10.1128/mBio.01193-19.7 TABLE S5 Ingenuity canonical pathway analysis of host differentially expressed genes. Download Table S5, XLSX file, 0.1 MB. Copyright © 2019 Griesenauer et al. 2019 Griesenauer et al. This content is distributed under the terms of the Creative Commons Attribution 4.0 International license.

10.1128/mBio.01193-19.8 TABLE S6 Ingenuity upstream regulatory analysis of host differentially expressed genes. Download Table S6, XLSX file, 0.1 MB. Copyright © 2019 Griesenauer et al. 2019 Griesenauer et al. This content is distributed under the terms of the Creative Commons Attribution 4.0 International license.

### Determination of an interaction network.

**FIG 4:**
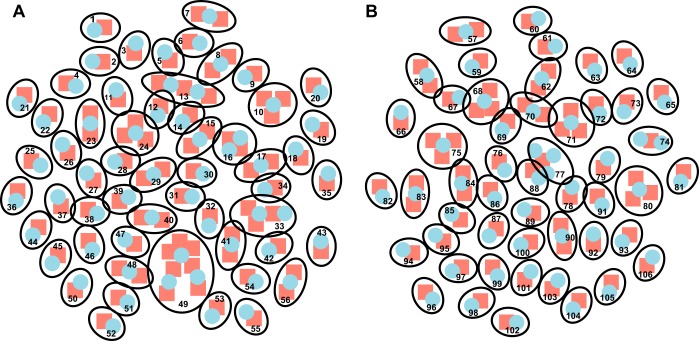
Bipartite network connecting the bacterial (blue circles) and host (red squares) gene pairs. Each node is outlined in black and numbered. (A) Genes that were positively correlated (*r* > 0.8). (B) Genes that were negatively correlated (*r* = less than −0.8). Lists of the genes corresponding to each node can be found in Table S7 for positive correlations and Table S8 for negative correlations.

We next asked if the changes in the levels of H. ducreyi and human gene expression were correlated. We calculated log_2_ ratios from normalized counts-per-million values for infected versus wounded sites for human genes and infected sites versus the inocula for H. ducreyi genes and generated an unbiased bipartite network connecting bacterial and host gene pairs that are statistically associated with one another. Given the sample size (*n* = 4 pairs), we used a stringent *P* cutoff value of *<*0.0002 and stringent *r* cutoff values of greater than 0.8 for positive interactions or and less than −0.8 for negative interactions. Using this approach, we identified 56 positively correlating networks and 50 negatively correlating networks containing 81 host and 61 bacterial DEGs and 65 host and 53 bacterial DEGs, respectively (Fig. 4; see also Tables S7 and S8). Multiple positively correlating networks contained H. ducreyi genes involved in anaerobic metabolism and human genes involved in the immune response, suggesting that H. ducreyi was responding to the metabolic niche shaped by the host response. For example, the H. ducreyi gene *napD*, which encodes a chaperone protein for *napA* (36) and is involved in anaerobic metabolism, correlated positively with the host genes *NFKB1* and *TNFAIP6*, which code for part of the NF-κB complex and a tumor necrosis family member, respectively, and are both involved in promoting an immune response. As well, the H. ducreyi gene *satB*, which encodes an integral membrane transporter for sialic acid (37), correlated positively with the host gene *FCAR*, which is found on myeloid cells and interacts with IgA to trigger various innate immune defenses.

10.1128/mBio.01193-19.9 TABLE S7 Positively correlating H. ducreyi and human differentially expressed genes. Download Table S7, XLSX file, 0.02 MB. Copyright © 2019 Griesenauer et al. 2019 Griesenauer et al. This content is distributed under the terms of the Creative Commons Attribution 4.0 International license.

10.1128/mBio.01193-19.10 TABLE S8 Negatively correlating H. ducreyi and human differentially expressed genes. Download Table S8, XLSX file, 0.02 MB. Copyright © 2019 Griesenauer et al. 2019 Griesenauer et al. This content is distributed under the terms of the Creative Commons Attribution 4.0 International license.

### Metabolomics studies.

**FIG 5:**
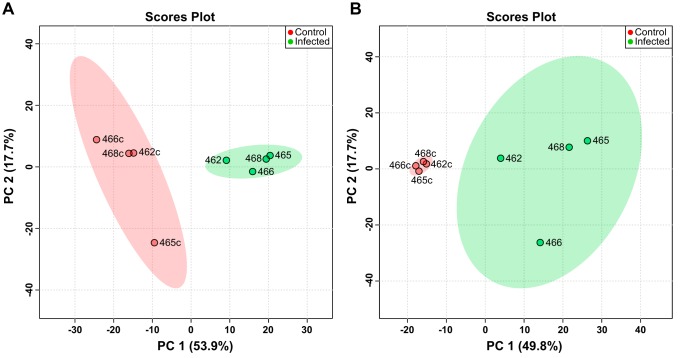
Principal-component analyses of (A) positive-ion and (B) negative-ion profiles from infected (green) and control (c) (red) samples from the volunteers (indicated by number).

We next asked which metabolites were enriched or diminished in infected versus wounded samples. As we did not have prior knowledge about the metabolites in an infected site, we took an untargeted approach. Principal-component analysis of both positive and negative ions showed clear separation between the infected and wounded samples, demonstrating that infection changed the metabolite composition in the skin (Fig. 5). To establish networks or pathways that were overrepresented or underrepresented in the infected versus wounded samples, we used Mummichog 2.0.6 (http://mummichog.org/). The top-scoring positive-ion pathway enriched in infected samples was the ascorbate and aldarate metabolism pathway (Table 3), which correlates with our H. ducreyi transcriptional data. Other enriched positive-ion pathways included linoleate, prostaglandin, and glutamate metabolism pathways, which play roles in innate immunity and lipid metabolism. Negative-ion pathway enrichment included many glycosphingolipid metabolism metabolites (Table 4). Glycosphingolipids are also involved in host-pathogen interactions and the immune response. Due to an insufficient number of overlapping samples, we were unable to formally integrate the transcriptomic data with the metabolomic data.

**TABLE 3:** Positive-ion pathways enriched in infected versus wounded sites

Pathway	No. of enriched metabolites	No. of metabolites in pathway	*P* value
Ascorbate and aldarate metabolism	3	4	1.43E−03
Linoleate metabolism	5	12	2.35E−03
Prostaglandin formation from arachidonate	5	13	4.03E−03
Glutamate metabolism	2	2	4.62E−03
Arachidonic acid metabolism	4	10	8.99E−03
Leukotriene metabolism	5	15	9.66E−03
Glutathione metabolism	2	3	1.32E−02
Anti-inflammatory metabolites from eicosapentaenoic acid	2	4	2.50E−02
Androgen and estrogen biosynthesis	2	4	2.50E−02
Carnitine shuttle	2	4	2.50E−02
Glycosphingolipid biosynthesis—globoseries	2	5	4.08E−02
Prostaglandin formation from dihomo gama-linoleic acid	1	1	4.33E−02

**TABLE 4:** Negative-ion pathways enriched in infected versus wounded sites

Pathway	No. of enriched metabolites	No. of metabolites in pathway	*P* value
Linoleate metabolism	9	21	6.30E−03
O-Glycan biosynthesis	6	11	7.23E−03
Keratan sulfate biosynthesis	6	11	7.23E−03
Glycosphingolipid biosynthesis—ganglioseries	6	12	1.10E−02
Blood group biosynthesis	6	12	1.10E−02
Glycosphingolipid biosynthesis—globoseries	6	12	1.10E−02
Glycosphingolipid biosynthesis—lactoseries	6	12	1.10E−02
Glycosphingolipid biosynthesis—neolactoseries	6	12	1.10E−02
Proteoglycan biosynthesis	5	10	1.75E−02
Glycosphingolipid metabolism	6	14	2.09E−02
Glutamate metabolism	3	5	3.25E−02
Keratan sulfate degradation	3	5	3.25E−02
Glutathione metabolism	3	5	3.25E−02
N-Glycan biosynthesis	6	17	4.32E−02

## DISCUSSION

The ability of a pathogen to adapt to stress caused by the host immune response is critical for the pathogen's survival. We have previously shown that expression of at least 18 genes or operons in the extracellular bacterium H. ducreyi is required for virulence of this pathogen in humans (35, 38–46). Comparison of transcripts from infected human sites to transcripts from mid-log-phase organisms suggested that upregulation of bacterial genes required for adaptation to nutrient stress and anaerobiosis was also involved in bacterial survival in humans (28). However, those previous studies did not address whether differential expression of human host genes or metabolites correlated with the expression of bacterial virulence determinants or differential expression of H. ducreyi genes. In this study, we identified host genes and metabolites that were associated with bacterial gene expression, uncovering how this pathogen and the human host interact.

To define an interactome between H. ducreyi and the human host, we performed RNA-seq on the inocula and infected and wounded tissue to identify differentially expressed H. ducreyi and host genes. We determined an interactome from differentially expressed H. ducreyi and host genes that both positively and negatively correlated with each other using an unbiased approach that did not require prior knowledge of gene function (3). We found multiple positively correlated networks containing H. ducreyi genes involved with anaerobic metabolism or acquisition of alternative (nonglucose) carbon sources and host genes involved in the immune response. These results are in accordance with our previous data (28) showing that anaerobic metabolism and alternative carbon uptake genes are upregulated in H. ducreyi*-*infected human pustules compared to *in vitro*-grown organisms and suggest that the immune response is driving this adaptation.

As most of the recent studies of nutritional immunity and virulence have focused on intracellular pathogens (47), less is known with respect to the manner in which extracellular bacteria exploit their host niche. Due to its net energy yield, glucose is among the nutrients most highly sought after by pathogens; many intracellular pathogens, such as Salmonella enterica and Brucella abortus, have developed mechanisms to steal glucose from within a host cell. Extracellular pathogens must use whatever nutrients are available in the environment outside the cell, which can vary greatly depending on the location of the infection and the influence of the immune response on nutrient access. Since H. ducreyi infects the skin, which contains high levels of glucose transporter 1 and hypoxia inducible factor-1 (48), and since the immune response promotes glucose consumption and hypoxia, H. ducreyi must find nonglucose sources of nutrients in an anaerobic environment to survive. Our data show that H. ducreyi upregulates the carbon starvation family member *cstA*, which is induced during glucose starvation (49), explaining why we observed upregulation of genes involved in the uptake of alternative carbon sources. We also found that genes involved in anaerobic metabolism were upregulated. Thus, we propose that changes to the local environment due to the immune response may be causing H. ducreyi to adapt by upregulating genes involved with nutrient acquisition and anaerobic metabolism, consistent with the idea of nutritional virulence (50).

Pathway analyses of the H. ducreyi gene sets showed that ascorbate and aldarate metabolism represented one of the two consistently upregulated pathways, suggesting that H. ducreyi is using l-ascorbate as a substitute for glucose as a carbon source. This pathway consists of the *ula* (utilization of l-ascorbic acid) genes, which have a variety of functions involved in ascorbic acid metabolism. UlaAB and UlaC take up and phosphorylate ascorbic acid, forming l-ascorbate-6-phosphate, which is converted to 3-keto-l-gulonate-6-phosphate by UlaG. UlaD, UlaE, and UlaF, which have decarboxylation and epimerase activities, converts this substrate to d-xylulose-5-phosphate, which is then metabolized by the pentose phosphate pathway (51). Correlating with the H. ducreyi transcriptional response, nontargeted metabolomics showed that ascorbate and aldarate metabolism is enriched in pustules. This enrichment suggests that ascorbic acid recycling is likely occurring in pustules, with neutrophils being the main source of l-ascorbate. Neutrophils migrate to the site of H. ducreyi infection, in part through TREM1 signaling, which was upregulated in our infected samples. As neutrophils attempt to clear infections, they take up l-ascorbate through a redox reaction termed ascorbic acid recycling (52). We found that glucose transporter 3, which helps with the recycling process through uptake of dehydroascorbic acid, the oxidized form of ascorbic acid (53), was upregulated in the infected samples. In an attempt to kill H. ducreyi, neutrophils undergoing NETosis are subject to membrane rupture (54) and could release their stored l-ascorbate, which is then scavenged by the invading H. ducreyi organisms. These data suggest that the H. ducreyi is responding to changes in the host metabolome caused by the host immune system.

Our data may have identified novel strategies for controlling infection that might be applicable to abscess forming organisms. For example, if uptake of l-ascorbate is required for H. ducreyi infection, it should be possible to target the *ula* pathway for novel therapeutics. We recently generated a *ulaABCD* mutant and compared its growth to strain 35000HP under anaerobic conditions in a supplemented GC broth containing either 0.1% dextrose or 1.5 mM ascorbic acid as an additional carbon source. Under anaerobic conditions, the mutant grew to the same extent as the wild type in the presence of dextrose but grew to levels significantly lower than those seen with the wild type in the presence of ascorbic acid. Under anaerobic conditions, 35000HP grew similarly in the presence of either dextrose or ascorbic acid, suggesting that both can serve as carbon sources for H. ducreyi (K. R. Fortney and S. M. Spinola, unpublished data). Given that an abscess is anaerobic, glucose poor, and enriched for ascorbic acid, we predict that the *ulaABCD* mutant may be attenuated *in vivo*. If this is confirmed, the *ula* pathway, which is present in other abscess-forming organisms such as Vibrio vulnificus, could serve as an antimicrobial target.

Of the 18 genes or operons known to be partially or fully required for pustule formation in humans, our study identified only 5 (*dsrA*, *flp-tad*, *hgbA*, *lspB-lspA2*, and *sapA*) that were upregulated (35, 42, 46, 55, 56). Other than *hgbA*, these genes are involved in the formation of microcolonies and resistance to complement-mediated killing, phagocytosis, and antimicrobial peptides. It also identified three genes (*pal*, *hfq*, and *fgbA*) that were downregulated, with *pal* having effects on structural integrity of the outer membrane, *hfq* having global effects on H. ducreyi gene expression, and *fgbA* involved in fibrin binding (40, 57, 58). Taken together, the data suggest that in a nutrient-poor environment, H. ducreyi upregulates only a few key virulence determinants needed to support its extracellular lifestyle.

Transcriptional profiles have been determined at sites of human infection for Mycobacterium tuberculosis and Staphylococcus aureus in previous studies (59, 60), while another study profiled biopsy samples of human gastric epithelial cells before and after antibiotic treatment for Helicobacter pylori infection (61); however, none of those studies determined the presence of an interaction network between the pathogen and the host. To our knowledge, dual RNA-seq has been performed for studies of pathogenic bacteria using only *in vitro* or murine models (1). Determining an interactome in naturally infected patients is difficult for several reasons, including the following: person-to-person variability in infecting bacterial strains, in host immune status, and in stage of disease; the lack of control samples that would allow determination of differential host and bacterial gene transcription *in vivo*; and the possibility of polymicrobial infections. An important strength of our study was that our model allowed us to infect healthy adults with a single bacterial strain to a defined stage of disease and provided controls for baseline gene transcription for both the bacterium and the host.

Small RNAs have major roles in the virulence of several Gram-negative bacteria such as Escherichia coli, Helicobacter pylori, and Vibrio cholerae (62–64). Results of dual RNA-seq analysis of *Salmonella*-infected HeLa cells showed that bacterial small RNAs regulate important functions in intracellular survival and manipulate host pathways to promote replication (3, 5, 65). Our RNA isolation procedures excluded transcripts that were <200 bp in size, which prevented us from examining differential regulation of host and bacterial small regulatory RNAs (66, 67). Because the infectious dose of H. ducreyi is low and the bacteria replicate to a mean of only 1.6 × 10^5^ ± 3.4 × 10^5^ (range, 10^2^ to 10^6^) CFU in endpoint pustules (68) (data not shown), we did not examine earlier time points of infection. In addition, for safety reasons, our protocols preclude us from infecting volunteers to the ulcerative stage. Thus, we did not do a time course study; such a study could help establish causality between differential regulation of a bacterial factor and the host response. Our study also did not address the cellular source of the host DEGs and metabolites we found in the pustules.

**FIG 6:**
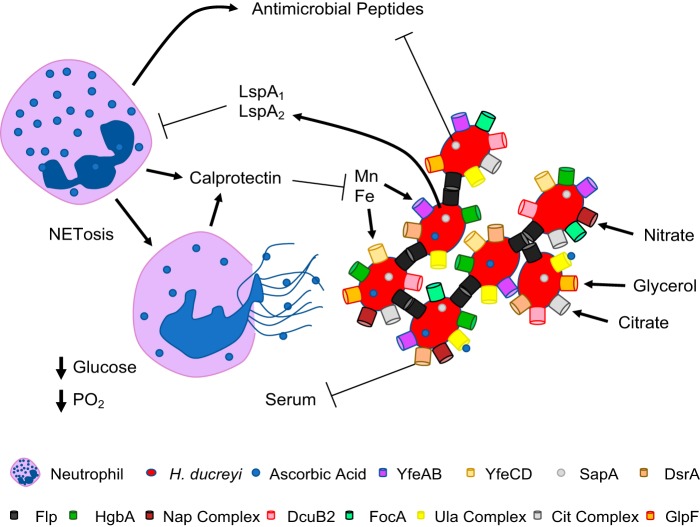
Model of major interactions between H. ducreyi and the human host. During infection, serum transudates into the wounds and the host immune system creates a microenvironment marked by low levels of glucose and oxygen (PO_2_). Neutrophils attempt to phagocytose H. ducreyi but are thwarted by the antiphagocytic proteins LspA_1_ and LspA_2._ Neutrophils likely undergoing NETosis release of calprotectin, antimicrobial peptides, and ascorbic acid. In response, H. ducreyi changes its gene transcription behavior to exploit the host microenvironment. H. ducreyi combats calprotectin by upregulating *yfeAB* and *yfeCD*, encoding transporters for manganese and iron. A few virulence factors required for human infection are upregulated, including LspA_1_ and LspA_2_; SapA, which transports antimicrobial peptides to the cytoplasm for degradation; DsrA, which prevents complement-mediated killing; the Flp proteins, which foster microcolony formation; and HgbA, which is responsible for hemoglobin uptake. Upregulation of the *nap* operon, *dcuB2*, and *focA* is consistent with adaptation to anaerobiosis. Lacking glucose, H. ducreyi acquires other carbon sources such as ascorbic acid, citrate, and glycerol by upregulating the *ula*, *cit*, and *glpF* operons or genes, respectively. Metabolomic data suggest that ascorbic acid may be the most abundant alternative carbon source at infected sites.

In summary, our data show that determination of an interactome between a bacterium and human host at the site of infection is feasible using dual RNA-seq. In the case of H. ducreyi infection, upregulation of host genes involved in the immune response strongly correlated with upregulation of bacterial genes involved with nutrient uptake, utilization of the alternative carbon source ascorbate, and adaptation to anaerobiosis, suggesting that H. ducreyi is adapting its gene transcription to its host environment (Fig. 6). Future studies will include deciphering which of the H. ducreyi genes that are necessary for adaptation to the host environment are required for virulence, further correlating gene expression with metabolites, and performing single-cell RNA-seq to determine the cellular sources of differentially expressed human genes.

## MATERIALS AND METHODS

### Bacterial strain and culture conditions.

The H. ducreyi strain utilized in this study was 35000HP, a human-passaged variant of strain 35000 (69). H. ducreyi was routinely grown on chocolate agar plates supplemented with 1% IsoVitaleX in the presence of 5% CO_2_. For the human challenge trials, H. ducreyi was grown to mid-log phase in a proteose peptone broth-based medium with 1% IsoVitaleX, 5% heat-inactivated fetal calf serum, and 50 μg/ml hemin (42). All cultures were grown at 33°C.

### Ethics statement.

Written informed consent was obtained from all the participants before enrollment in the study. The study was approved by the Institutional Review Board of Indiana University.

### Human inoculation experiments.

Stocks of 35000HP and the inocula were prepared under good laboratory practices and good manufacturing practice protocols according to U.S. Food and Drug Administration guidelines under BB-IND no. 13064. Methods for preparation and inoculation of the bacteria, determination of the estimated delivered dose, biopsy sampling, clinical observations, and antibiotic treatment of the volunteers were performed exactly as described previously (18). Clinical endpoints included resolution of infection at all sites, the development of a painful pustule at any site, or 14 days of observation (18).

### RNA isolation and quality assessment.

Dedicated lots of reagents were used for all specimens. Biopsy samples and an aliquot of the inocula were placed in 2 ml RNAlater, incubated for 30 min at room temperature, and stored in RNAlater for 1 day at −80°C. Using a mini-Beadbeater (Biospec Products), we next homogenized the samples and extracted total RNA using a RNeasy fibrous tissue minikit according to the manufacturer’s instructions along with addition of lysozyme (20 μg/ml) at the proteinase K step. The RNA was treated twice with Turbo DNA-free DNase (Ambion) following the manufacturer’s instructions. RNA concentrations and integrities were measured using a model P330 NanoPhotometer (Implen). RNA samples were stored at −80°C until all samples were ready for sequencing.

### mRNA enrichment.

We removed 23S, 16S, and 5S rRNA before RNA-seq by the use of a Ribo-Zero Gold rRNA removal kit (Epidemiology) (Epicentre Biotechnologies) following the manufacturer’s instructions and confirmed the removal of each using an Agilent 2100 Bioanalyzer.

### RNA-seq library preparation and sequencing.

Twelve libraries (four mid-log-phase-growth H. ducreyi cultures, four infected sites, and four wounded sites) were constructed using a TruSeq stranded total RNA library kit (Illumina) following the manufacturer’s instructions. The libraries were sequenced on a Hi-Seq 4000 system (Illumina) for paired-end sequencing with read lengths of 75 bp using eight lanes of a single flow cell. Sequence reads were mapped to H. ducreyi and human genomes using the ASM794v1 (GenBank assembly accession no. GCA_000007945.1) and hg38 (GenBank accession assembly no. GCA_000001405.27) assemblies, respectively, and TopHat-Cufflinks. H. ducreyi reads that failed to map to any gene or mapped to multiple genes were removed before transcript analysis. All human reads that mapped to hg38 were retained. As the number of human reads was greater in the infected sites than in the wounded sites and as the number of H. ducreyi reads was greater in the inocula sites than in the infected sites, the reads were subsampled. The data from these RNA-seq experiments were deposited at the NCBI Gene Expression Omnibus (GEO) database (see below).

### Identification of differentially expressed genes (DEGs).

The Bioconductor package “edgeR” was used to determine differential expression of H. ducreyi and human genes (70). We first prefiltered the results by removing genes showing low levels of expression. Raw read counts were then normalized using trimmed means of M values. Multidimensional scaling plots (MDS) for H. ducreyi and human transcriptomes were then generated. We used the “vegan” package to perform permutational multivariate analysis of variance of each MDS plot (71). Differential expression of genes between paired groups was determined using a Cox-Reid profile-adjusted likelihood method to fit the data into a negative-binomial generalized linear model to estimate dispersions followed by the quasilikelihood F-test (qlf) to test for differential expression. The blocking factor used corresponded to the volunteers. Differential expression was defined using absolute log_2_ fold change values of ≥1 and false-discovery-rate values of <0.01. Pearson coefficients were determined to test for correlations of bacterial and host gene expression between pairs of volunteers and for correlations of bacterial gene expression between pairs of the inocula.

### qRT-PCR analysis.

We performed qRT-PCR on H. ducreyi genes using a QuantiTect SYBR green RT-PCR kit (Qiagen) on an ep realplex4 Mastercycler (Eppendorf). Primer pairs are listed in Table S2 in the supplemental material. We normalized expression levels to that of *dnaE*, which was expressed similarly between infected and mid-log-phase H. ducreyi bacteria. Because we used all the wound control RNA for RNA-seq, we could not perform qRT-PCR analyses on the host transcripts.

### Enrichment analyses.

H. ducreyi DEGs were functionally classified using KEGG terms (72). We manually curated a gene set list based solely on KEGG terms with single genes that were possibly a part of multiple gene sets; 141 gene sets in all were created (Table S3 in supplemental material). Human genes were separated by GO terms using the gene matrix from MSigDB (c5.all.v6.2.symbols). Multiple tests, including preranked GSEA and the CERNO test, a variant of the Mann-Whitney U test that is better for analysis of small sample sizes, were performed to determine which functional classifications for H. ducreyi and human were differentially expressed (73). The R package tmod was used for running the CERNO test (74). H. ducreyi and human genes for GSEA and tmod were preranked by log fold change and prefiltered based on our differentially expressed gene criteria. Gene sets were considered to be statistically different only if the two testing methods agreed. For GSEA, statistically different functional groupings were classified as having a normalized enrichment score of ≥2 and a false-discovery rate (FDR [*q*]) of <0.01. Enrichment plots were checked for verification of leading edges of bacterial gene sets that contained <10 genes/set. For CERNO, statistically different functional groupings were classified as having an adjusted *P* value of <0.05.

### IPA analyses.

Ingenuity pathway analysis (IPA) software (Qiagen) was used for pathway and network analyses of the human transcriptome data (75). Canonical pathway analysis identified significantly altered pathways, and upstream regulator analysis identified upstream or downstream activation or inhibition of a pathway. Z-scores of more than 2 or less than −2 were considered matches. *P* values of <0.01 were considered statistically significant.

### Interactome network.

A generalized linear model was constructed using the fold changes in the host DEGs as dependent variables and the fold changes for bacterial DEGs as independent variables. Using log_2_ ratios of the host and bacterial DEGs, a bipartite network, using the R package “igraph” (76), was generated connecting the H. ducreyi and human gene pairs using cutoffs of an unadjusted *P* value of <0.0002 and Pearson coefficients of *r* greater than 0.8 for positive interactions and *r* less than −0.8 for negative interactions.

### Metabolomics.

Each biopsy pair (infected and wounded tissue from the same volunteer) was washed in phosphate-buffered saline (PBS), snap-frozen in liquid nitrogen, and stored at −80°C. The frozen tissue was ground into a powder in the presence of 80% methanol on dry ice; the supernatant was subjected to nano-liquid chromatography-mass spectrometry using a Sciex 5600 TripleTOF mass spectrometer to identify ions up to *m/z* 1,000. Ions were aligned across all samples using XCMS Online (https://xcmsonline.scripps.edu) and peak areas recorded. Peak areas of the samples were normalized for total ion content, and Pareto scaling was applied. MetaboAnalyst 4.0 (77) and Mummichog 2.0.6 (78) were used to identify groups of positive and negative ions that were enriched or diminished and to establish networks or pathways that were overrepresented or underrepresented in infected and wounded samples, respectively. *P* values of <0.05 were considered statistically significant.

### Data availability.

The data from these RNA-seq experiments were deposited at the NCBI Gene Expression Omnibus (GEO) database (see below) under accession number GSE130901.
