# Supplementary material for: Determination of an Interaction Network between an Extracellular Bacterial Pathogen and the Human Host
Source: mBio. 2019 Jun 18;10(3):e01193-19. doi: 10.1128/mBio.01193-19 (PMC6581864; doi:10.1128/mBio.01193-19)
Supplement: FIG S1 [file mBio.01193-19-sf001.pdf]

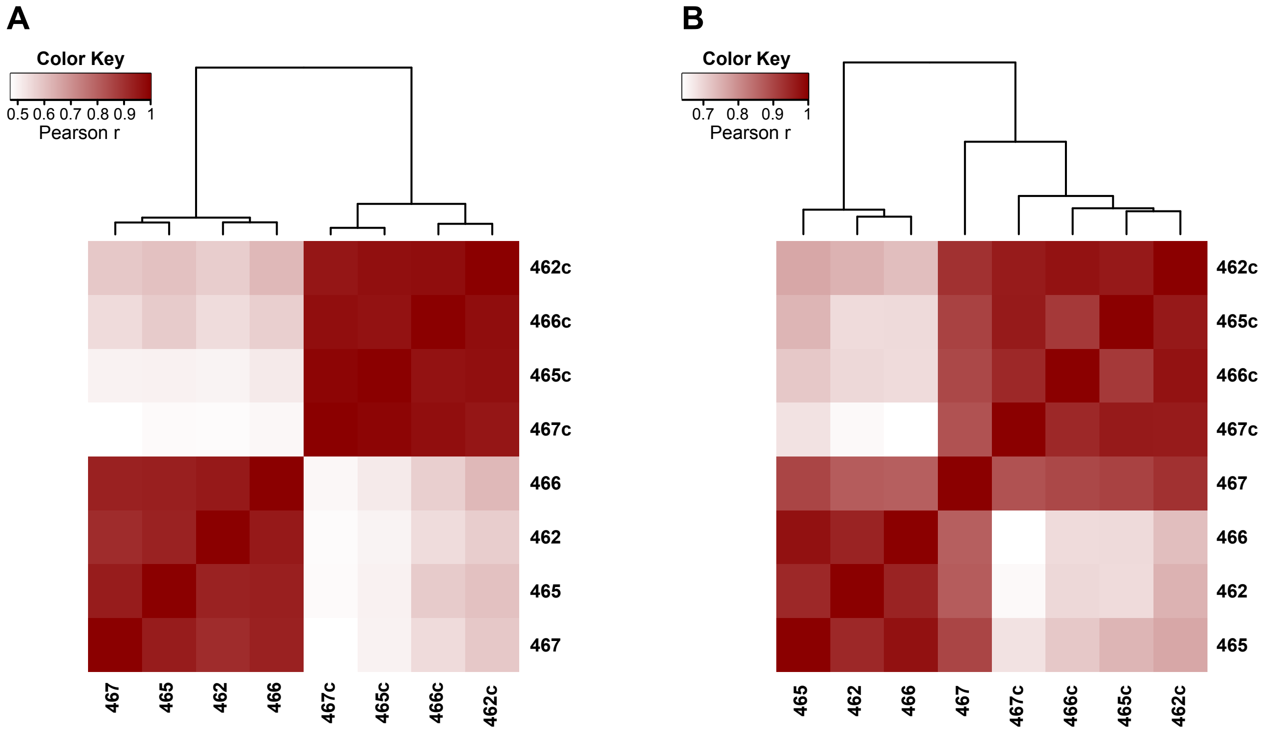

FIG S1 Heatmaps of (A) *H. ducreyi* and (B) human genes showing Pearson correlation coefficients (r) between volunteers at infected sites (462, 465, 466, and 467) and (A) inocula or (B) wounded sites (462c, 465c, 466c, and 467c), respectively. Pearson correlation coefficients were calculated after removal of low-expressing genes and transformation of counts to CPM (counts per million).
