# Supplementary material for: Determination of an Interaction Network between an Extracellular Bacterial Pathogen and the Human Host
Source: mBio. 2019 Jun 18;10(3):e01193-19. doi: 10.1128/mBio.01193-19 (PMC6581864; doi:10.1128/mBio.01193-19)
Supplement: TABLE S2 [file mBio.01193-19-st002.docx]

| **TABLE S2** *H. ducreyi* primers used in this study | |
| --- | --- |
| Primer Name | 5’ to 3’ sequence |
| *napD* F | GCCAAATTACCCCAAGTGAA |
| *napD* R | CGCCGTTGATCTCTTTTAGC |
| *metK* F | CACGTGTAGCGTGTGAAACC |
| *metK* R | GGCACCTTGTGCTAATGGAT |
| *satB* F | CTGCCCTTTATCGTGATCGT |
| *satB* R | AAGCGGTACAAAACCACCTG |
| *citC* F | TGGCATTGCTGATTTATCCA |
| *citC* R | GTTCCGTTCCGATAAAACGA |
| *hfq* F | TCGAGAGCGTATTCCCGTCTCAAT |
| *hfq* R | TGTACGGCTTGTTGAGGAGCTTGT |
| *pal* F | GATGCTATGGCGGTTAATCA |
| *pal* R | GTTCATCCGCATGTCCTGTA |
| *ulaG* F | AGAACAAGTACCGGCCAATAA |
| *ulaG* R | GCCTCGACCACACCATAAA |
| *ulaAB* F | TAATGGCTGGATGGGAATGG |
| *ulaAB* R | GAGGCTCACCAAATCCGTATAA |
| *dnaE* F | AACGTTACCTTCAGCAAGCGGTTC |
| *dnaE* R | GGCGTTTGGGATCGTCGAGTGTAT |
