# Supplementary material for: Determination of an Interaction Network between an Extracellular Bacterial Pathogen and the Human Host
Source: mBio. 2019 Jun 18;10(3):e01193-19. doi: 10.1128/mBio.01193-19 (PMC6581864; doi:10.1128/mBio.01193-19)
Supplement: FIG S2 [file mBio.01193-19-sf002.pdf]

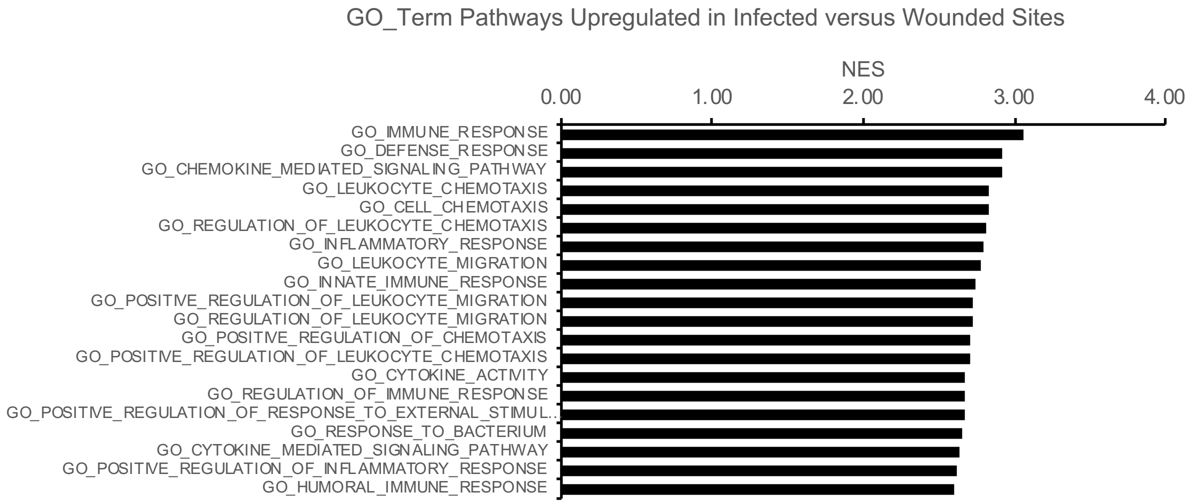
GO_Term Pathways Upregulated in Infected versus Wounded Sites
NES
0.00
1.00
2.00
3.00
4.00
GO_IMMUNE_RESPONSE
GO_DEFENSE_RESPONSE
GO_CHEMOKINE_MEDIATED_SIGNALING_PATHWAY
GO_LEUKOCYTE_CHEMOTAXIS
GO_CELL_CHEMOTAXIS
GO_REGULATION_OF_LEUKOCYTE_CHEMOTAXIS
GO_INFLAMMATORY_RESPONSE
GO_LEUKOCYTE_MIGRATION
GO_INNATE_IMMUNE_RESPONSE
GO_POSITIVE_REGULATION_OF_LEUKOCYTE_MIGRATION
GO_REGULATION_OF_LEUKOCYTE_MIGRATION
GO_POSITIVE_REGULATION_OF_CHEMOTAXIS
GO_POSITIVE_REGULATION_OF_LEUKOCYTE_CHEMOTAXIS
GO_CYTOKINE_ACTIVITY
GO_REGULATION_OF_IMMUNE_RESPONSE
GO_POSITIVE_REGULATION_OF_RESPONSE_TO_EXTERNAL_STIMUL...
GO_RESPONSE_TO_BACTERIUM
GO_CYTOKINE_MEDIATED_SIGNALING_PATHWAY
GO_POSITIVE_REGULATION_OF_INFLAMMATORY_RESPONSE
GO_HUMORAL_IMMUNE_RESPONSE

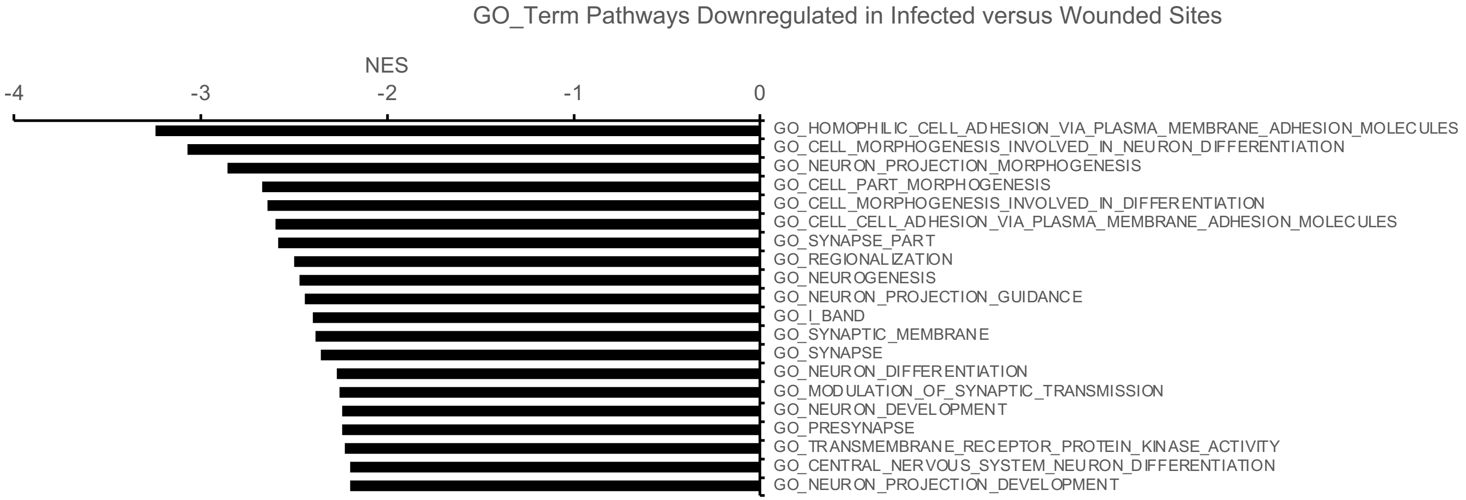
GO_Term Pathways Downregulated in Infected versus Wounded Sites
NES
-4
-3
-2
-1
0
GO_HOMOPHILIC_CELL_ADHESION_VIA_PLASMA_MEMBRANE_ADHESION_MOLECULES
GO_CELL_MORPHOGENESIS_INVOLVED_IN_NEURON_DIFFERENTIATION
GO_NEURON_PROJECTION_MORPHOGENESIS
GO_CELL_PART_MORPHOGENESIS
GO_CELL_MORPHOGENESIS_INVOLVED_IN_DIFFERENTIATION
GO_CELL_CELL_ADHESION_VIA_PLASMA_MEMBRANE_ADHESION_MOLECULES
GO_SYNAPSE_PART
GO_REGIONALIZATION
GO_NEUROGENESIS
GO_NEURON_PROJECTION_GUIDANCE
GO_I_BAND
GO_SYNAPTIC_MEMBRANE
GO_SYNAPSE
GO_NEURON_DIFFERENTIATION
GO_MODULATION_OF_SYNAPTIC_TRANSMISSION
GO_NEURON_DEVELOPMENT
GO_PRESYNAPSE
GO_TRANSMEMBRANE_RECEPTOR_PROTEIN_KINASE_ACTIVITY
GO_CENTRAL_NERVOUS_SYSTEM_NEURON_DIFFERENTIATION
GO_NEURON_PROJECTION_DEVELOPMENT
